# Supplementary material for: Exploring the Use of Fruit Callus Culture as a Model System to Study Color Development and Cell Wall Remodeling during Strawberry Fruit Ripening
Source: Plants (Basel). 2020 Jun 27;9(7):805. doi: 10.3390/plants9070805 (PMC7412483; doi:10.3390/plants9070805)

Exploring the use of fruit callus culture as a model system to study color development and cell wall remodeling during strawberry fruit ripening

Pablo Ric-Varas, Marta Barceló, Juan A. Rivera, Sergio Cerezo, Antonio J. Matas, Julia Schückel, J. Paul Knox, Sara Posé, Fernando Pliego-Alfaro, José A. Mercado

Supplementary Material

Supplementary Table S1. List of monoclonal antibodies used in the carbohydrate microarray and the epitopes recognized by each mAb.

Antibody	Cell wall epitope	Reference
LM18	Partially Me-HG / no ester	Verhertbruggen et al. (2009) Carbohydr. Res. 344, 1858
LM19	Partially Me-HG / no ester	Verhertbruggen et al. (2009) Carbohydr. Res. 344, 1858
JIM5	Partially Me-HG / no ester	Knox et al. (1990) Planta 181, 512-521
JIM7	Partially Me-HG	Knox et al. (1990) Planta 181, 512-521
LM5	(1→4)-β-D-galactan	Jones et al. (1997) Plant Physiol. 113, 1405-1412
LM6-M	(1→5)-α-L-arabinan	Cornuault et al. (2017) BiorXiv: doi.org/10.1101/161604
INRA-RU1	$[\rightarrow 2)$-α-L-rhamnose-(1→4)-α-D-galacturonic acid-$(1\rightarrow]_7$	Ralet et al. (2010) Planta 231, 1373-1383
INRA-RU2	$[\rightarrow 2)$-α-L-rhamnose-(1→4)-α-D-galacturonic acid-$(1\rightarrow]_7$	Ralet et al. (2010) Planta 231, 1373-1383
LM13	Linearised (1→5)-α-L-arabinan	Moller et al. (2008) Glycoconjugate J. 25, 37-48
LM26	Branched galactan	Torode et al. (2018) Plant Physiol. 176, 1547-1558
LM15	Xyloglucan (XXXG motif)	Marcus et al. (2008) BMC Plant Biol. 8, 60
LM25	XXXG/galactosylated xyloglucan	Pedersen et al. (2012) J. Biol. Chem. 287, 39429-39438
LM11	(1→4)-β-D-xylan / arabinoxylan	McCartney et al. (2005) J. Histochem Cytochem 53, 543
LM23	Non-acetylated xylosyl	Manabe et al. (2011) Plant Physiol. 155, 1068-1078
LM28	Glucuronoxylan	Cornuault et al. (2015) Planta 242, 1321-1334
LM1	Extensin	Smallwood et al. (1995) Planta 196, 510-522
JIM11	Extensin	Smallwood et al. (1994) Plant J. 5, 237-246
JIM20	Extensin	Smallwood et al. (1994) Plant J. 5, 237-246
JIM13	AGP glycan	Knox, et al. (1991) Plant J. 1, 317-326
LM21	Heteromannan	Marcus et al. (2010) Plant J. 64, 191-203
LM27	Grass heteroxylan	Cornuault et al. (2015) Planta 242, 1321-1334

Supplementary Figure S1. Variables factor map obtained in the PCA analysis of carbohydrate microarray data.

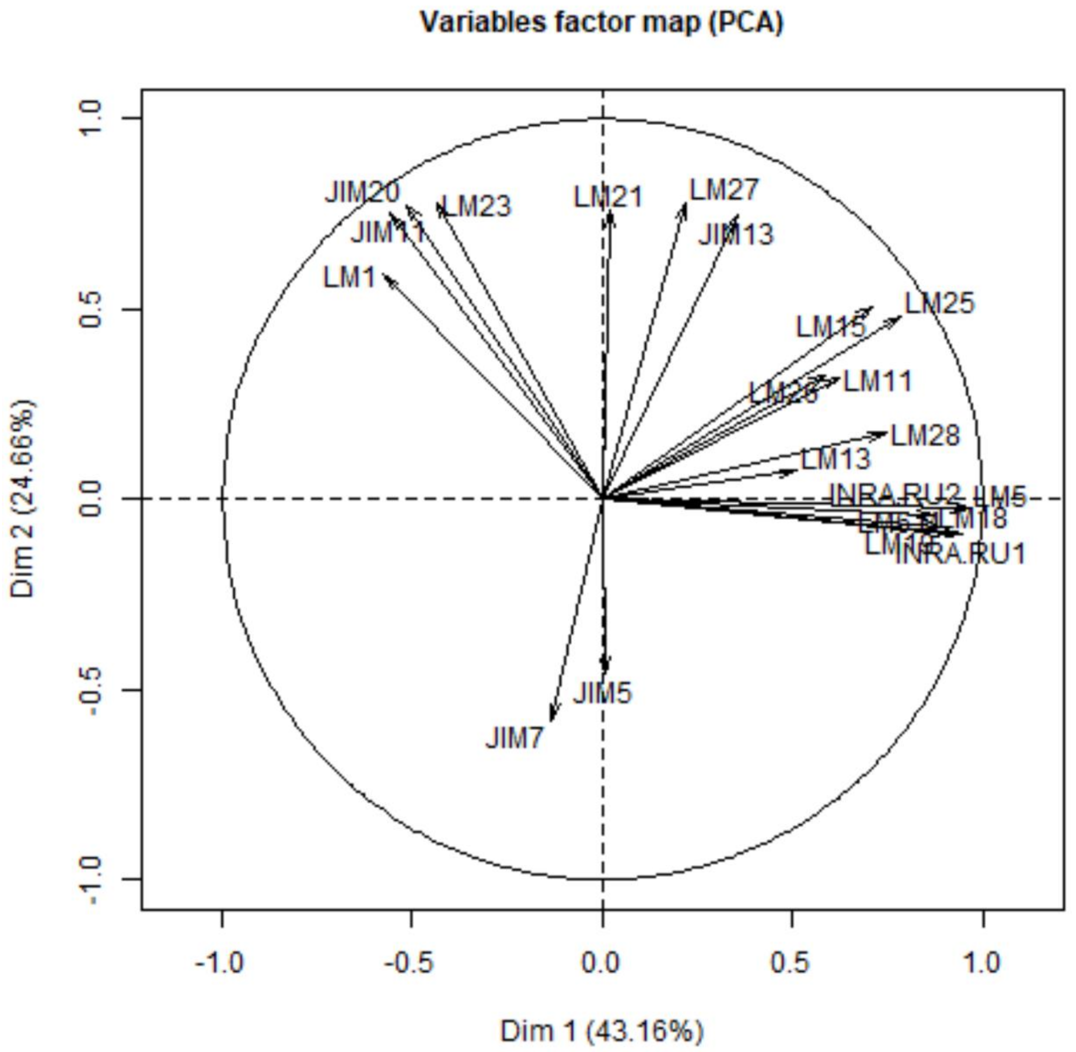

Supplement: Supplementary file 1 [file plants-09-00805-s001.pdf]
